# The effect of multidrug exposure on neurological manifestations in carbamazepine intoxication: a nested case-control study

**DOI:** 10.1186/s40360-020-00425-2

**Published:** 2020-06-29

**Authors:** Ayala Hirsch, Maor Wanounou, Amichai Perlman, Bruria Hirsh-Raccah, Mordechai Muszkat

**Affiliations:** 1 Department of Internal Medicine, Hadassah-Hebrew University Medical School, Mt Scopus, POB 24035, Ein Kerem, 91240 Jerusalem, Israel; 2 Department of Cardiology, Hadassah-Hebrew University Medical School, Ein Kerem, Jerusalem, Israel; 3 Division of Clinical Pharmacy, Institute for Drug Research, School of Pharmacy, Faculty of Medicine, Hebrew University of Jerusalem, Jerusalem, Israel

**Keywords:** Carbamazepine, Intoxication, Concentration level, Concomitant medication, Benzodiazepines

## Abstract

**Background:**

In acute intoxication, carbamazepine concentration above 40 mcg/ml is associated with a risk of severe neurological consequences, including depressed consciousness, respiratory depression, cardiac conduction disorders, seizures, and death. Carbamazepine intoxication is often associated with the use of concomitant medications. However, the effect of exposure to other central-nervous-system (CNS) acting medications on the neurological manifestations of carbamazepine toxicity has not been evaluated.

**Objective:**

To examine the effect of exposure to CNS-acting medications on the neurological effects of carbamazepine toxicity.

**Methods:**

A retrospective nested case-control study of all patients > 18 years of age, with at least one test of carbamazepine levels > 18 mcg/ml recorded at the Hadassah Hospital Central Laboratory, between the years 2004–2016. Sociodemographic and clinical data were collected from the computerized medical records, and the characteristics of patients with and without severe neurological symptoms of carbamazepine intoxication were compared.

**Results:**

Eighty patients were identified. In bivariate analyses, the odds of severe neurological symptoms was higher in patients with antidepressants use (odds ratio 8.7, 95% confidence interval: 1.8–41.2, *p* = 0.007), benzodiazepines use (8.6, 2.0–37.1, *p* = 0.004), and carbamazepine concentration above 30 mcg/ml (8.1, 1.9–33.3, *p* = 0.004).

Multivariate models demonstrated that antidepressants and benzodiazepines were associated with severe neurological manifestations during carbamazepine intoxication, independently of carbamazepine concentration over 30 mcg/ml.

ICU admission was associated in multivariate analysis with antidepressants (but not benzodiazepines) use, and with carbamazepine levels > 30 mcg/ml.

**Conclusions:**

Among patients with carbamazepine intoxication, severe neurological symptoms are associated with exposure to benzodiazepines or antidepressants and with carbamazepine levels higher than 30 mcg/ml.

## Background

Carbamazepine is widely used for the treatment of epilepsy, affective disorders, trigeminal neuralgia, and other conditions. Carbamazepine’s action is attributed to various pharmacological mechanisms [1]. Carbamazepine stabilizes the inactivation state of sodium channels both in the central nervous system and in the heart, thereby reducing depolarization and decreasing glutamate release. This may explain the neurological and cardiac effects observed in overdose situations [2]. Carbamazepine has anticholinergic activity, and has a paradoxical effect on adenosine’s receptor: in a therapeutic dose, carbamazepine inhibits adenosine reuptake, thereby inhibiting glutamate release, while in overdose it has an antagonistic effect on adenosine receptor. This mechanism can explain the occurrence of seizures during carbamazepine overdose.

Therapeutic drug monitoring of carbamazepine is used in routine clinical practice.

During intoxication, measurement of carbamazepine concentration may have important implications for patient management, such as decision-making regarding ICU admissions. Generally, a correlation between drug concentration and clinical toxicity has been observed. However, the findings regarding the level associated with the risk for severe neurological manifestation have been inconsistent.

Carbamazepine concentrations above 40 mcg/ml are considered to be associated with the risk for severe clinical manifestations such as depressed consciousness, respiratory depression, cardiac conduction disorders, seizures, and death [3]. However, carbamazepine levels associated with neurological depression differ between the pediatric and adult populations. Children younger than 12 years may develop severe complications during carbamazepine intoxication at lower carbamazepine levels than adults [4] . In a study of 263 patients with carbamazepine intoxication, carbamazepine levels of 20–30 mcg/ml were associated with severe symptoms in 8% of adults and 43% of the children. At levels above 30 mcg/ml, only 33% of adults had severe neurological symptoms while 85% of children had such symptoms [5].

In a retrospective review of 28 adult cases with isolated carbamazepine poisoning, among patients with levels above 40 mcg/ml, 60% developed at least two of the following: seizures, coma, or respiratory depression. Only one subject had severe symptoms with carbamazepine levels lower than 40 mcg/ml [3]. However, severe symptoms have been reported with levels lower than 40 mcg/ml as well [6, 7].

Based on these data, clinical guidelines have suggested that the risk of severe symptoms of carbamazepine overdose is associated with levels greater than 40 mcg/ml^1^.

In clinical practice, carbamazepine intoxication often occurs in patients who are exposed to concomitant medications with central nervous system (CNS) effects, and suicidal mortality has been associated with multi-drug toxicity [8]. Nevertheless, most studies evaluating the relationship between carbamazepine concentrations and the manifestations of intoxication have not reported on the effect of concomitant CNS medications. Thus, we sought to examine the relationship between carbamazepine concentration and neurological symptoms in acute multi-drug toxicity in adults.

## Methods

We performed a retrospective nested case-control study of all patients with carbamazepine concentration measurements at the central laboratory of the Hadassah University Hospital, between the years 2004 and 2016. The study was approved and exempted from informed consent by the Hadassah Institutional Ethics Committee.

### Study population

The study population included men and women over 18 years of age in whom carbamazepine concentrations above 18 mcg/ml were measured at the central laboratory of the Hadassah University Hospital between the years 2004 and 2016.

Patients were excluded if carbamazepine levels were below 18 mcg/ml, based on previous literature [3], if their medical files were confidential, or if they had a non-pharmacological event that could affect neurological status. In patients with more than one measurement of carbamazepine concentration, the highest measurement was used.

### Determination of carbamazepine concentration

Patients’ carbamazepine blood concentration was tested at the central laboratory of the Hadassah University Hospital using fluorescence polarization with COBAS INTEGRA reagent system cassettes on COBAS INTEGRA 700 (Roche Diagnostics) [9] . The reagents, controls, and calibrators were obtained from Roche Diagnostics and were used according to the manufacturer’s instructions.

### Data collection

Sociodemographic and clinical data were anonymously retrieved from the computerized medical records, and the characteristics of patients with and without severe neurological manifestations of carbamazepine intoxication were compared. Data collected included: age, gender, background diseases, carbamazepine plasma concentration (mcg/ml), cause for ingestion (accidental, intentional, suicide attempt, therapeutic), carbamazepine daily dosage, other drugs used, as well as clinical findings including: neurological, respiratory, cardiovascular findings, ECG findings, ICU admission, treatment, and duration of hospitalization. All additional drugs were recorded using the ATC system [10].

A severe neurological presentation was defined as severely depressed consciousness i.e., impaired or no response to voice or pain, or the presence of stupor or coma.

Patients without severe neurologic manifestations included patients with no neurologic manifestations, patients with neurologic symptoms but without a change in the state of consciousness or patients with a mild change in the state of consciousness, such drowsiness, but with intact response to voice.

### Statistical analysis

Continuous variables were expressed as mean ± SEM and categorical variables as percentages. Patients with and without severe neurological presentation of carbamazepine intoxication were compared. The χ^2^ test was used for comparison of categorical variables, and t-test for continuous variables. Factors that were significantly associated with a severe neurological presentation were examined using multiple logistic regression. *P*-value < 0.05 was considered significant. All statistical analyses were performed using IBM-SPSS version 24.0 (IBM Corp., Armonk, NY, USA).

## Results

We identified 203 carbamazepine concentration tests from 136 different subjects with carbamazepine concentration greater than 18 mcg/ml during the period specified. Thirty-three of these 136 subjects were younger than 18 years, and 20 were missing essential information (i.e. information on background diseases and concomitant medications). Of the remaining 83 patients, three were excluded due to non-pharmacological acute events (including acute meningitis, hepatic encephalopathy, and head trauma) which had likely affected neurological presentation. Thus, a total of 80 patients were included in our analysis.

### Patient characteristics

The socio-demographic and clinical data of the 80 patients included in the analysis are shown in Table 1. The average age was 43.4 ± 1.96 years. Fifty-two percent were men. 67% were Jews and 31% Arabs. Before intoxication, carbamazepine was used chronically by 83% of the patients. The main indications for chronic use were epileptic seizures (63%) and affective disorder (34%). Carbamazepine concentration greater than 30 mcg/ml was found in 21.0% of patients, and 8.8% had carbamazepine concentration greater than 40 mcg/ml. Severe neurological symptoms were observed in 10 (12.5%) patients, while in 70 patients (87.5%) there were no severe symptoms of intoxication.

**Table 1:** Sociodemographic and clinical characteristics of patients with carbamazepine intoxication. Continuous variables are presented as average ± SEM, ordinal variables are presented as number and percentage

	All patients	Neurological Symptoms		
	(*n* = 80)	Non-Severe (*n* = 70)	Severe (*n* = 10)	*p-*value*
Age (Years)	43.4 ± 2.0	45.2 ± 2.1	30.0 ± 3.5	0.00
Gender (% female)	38 (47.5)	32 (45.7)	4 (40)	0.39
Ethnicity:				0.03
Arab	25 (31.3)	24 (34.3)	1 (10)	
Jews	54 (67.5)	46 (66.7)	8 (80)	
Others	1 (1.3)	0	1 (10)	
Smokers	6 (7.5)	6 (8.9)	0 (0)	0.196
Chronic Carbamazepine users	67 (83.8)	59 (84.3)	8 (80)	0.32
**Indications for carbamazepine:**				
Epileptic seizures	50 (62.5)	47 (67.1)	4 (40)	0.09
Psychiatric disorder	27 (33.7)	20 (28.5)	7 (70)	0.013
Neuropathic pain	4 (5)	4 (5.7)	0 (0)	0.29
Others^a^	5 (6.2)	5 (7.1)	0 (0)	

*P-value was calculated by t-tests for continuous variables and Chi-square test for ordinal variables

^a^Others- primary sclerosis, psychomotor retardation and accidental ingestion

### Clinical characteristics

**Fig. 1:**
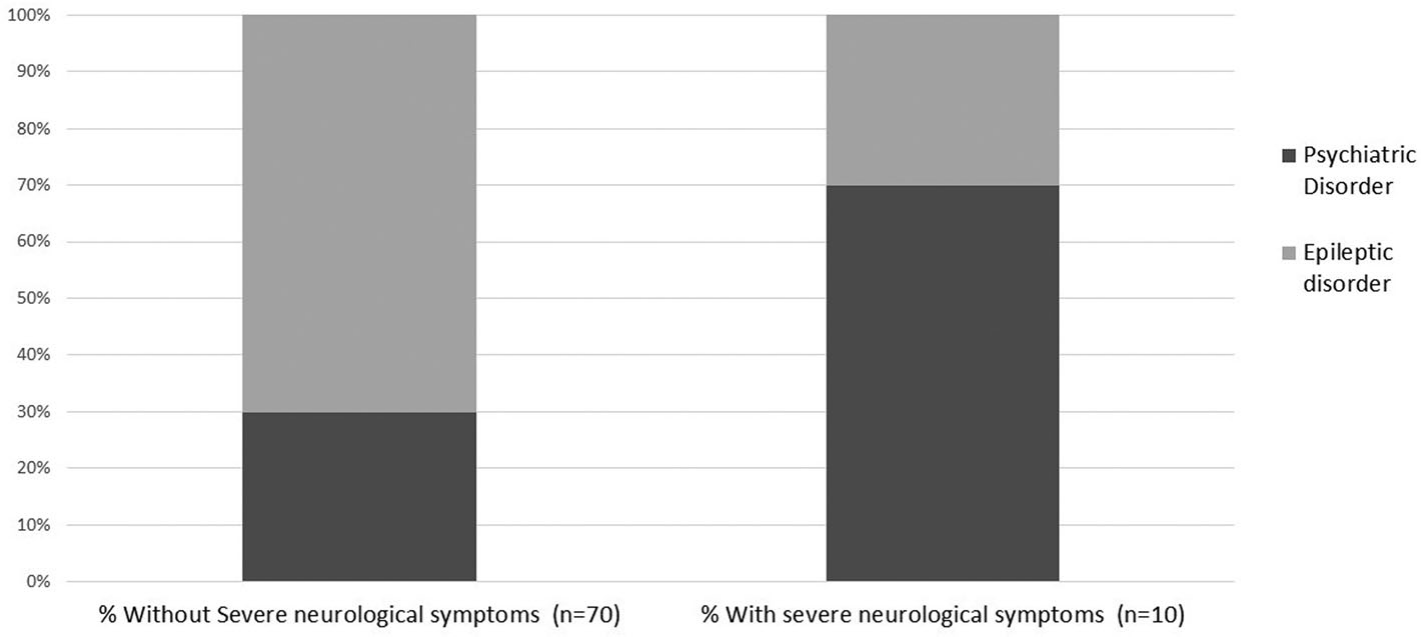
Carbamazepine intoxication- clinical characteristics of study subjects with and without severe neurological symptoms. *all *p*-value< 0.05

Background characteristics of patients with and without severe neurological symptoms of carbamazepine intoxication are shown in Table 1. Carbamazepine was prescribed for a psychiatric disorder in 70% of patients with severe neurologic symptoms, as compared to 29% among those without severe neurologic manifestations (*p* = 0.013) (Fig. 1). In contrast, the predominant indication for carbamazepine in patients without severe neurological symptoms was epileptic seizures (68%) (*p* = 0.09) (Table 1). No other differences in background diseases or baseline characteristics were found between the groups. There was no significant difference in the rate of chronic carbamazepine users between those who suffered severe neurologic symptoms and those who did not.

Other medications in addition to carbamazepine that affect the central nervous system were used in 66% of all patients: Thirty-five percent were treated with another antiepileptic drug, and 27% were treated with a benzodiazepine (Table 1).

### Clinical presentation of carbamazepine intoxication

The clinical manifestations of carbamazepine intoxication are presented in Table 2. A suicide attempt was observed in 36% of patients. Thirty-two patients (40%) did not have neurological symptoms, 38 patients (47.5%) had non-severe symptoms and 10 (12.5%) had severe neurological symptoms.

**Table 2:** Clinical presentation, management and clinical outcomes of patients with carbamazepine intoxication. Continuous variables are presented as average ± SEM, ordinal variables are presented as number and percentage

	All patients	Neurological Symptoms		*P* value
	(n = 80)	Non-severe (n = 70)	Severe (n = 10)	
Neurological Presentation:				
No symptoms	11 (13.8)	11 (15.9)	0 (0)	
Drowsiness	58 (72.5)	58 (84.1)	0 (0)	
No response to voice/pain	10 (12.50)	0 (0)	10 (100)	
CBZ level (Average, mcg/mL)	26.3 ± 1.8	24.4 ± 1.2	40.0 ± 11.1	0.19
CBZ level (mcg/ml):				0.03
18–20	41 (51.2)	38 (54.3)	3 (30)	
20–30	22 (27.5)	21 (30)	1 (10)	
30–40	10 (12.50)	6 (8.6)	4 (40)	
> 40	7 (8.8)	5 (7.1)	2 (20)	
Carbamazepine dose (acute ingestion, mg)	3623.1 ± 568.0 (*n* = 65)	3338.3 ± 581.9 (*n* = 58)	5982.9 ± 2064.9 (n = 7)	0.15
Suicide attempt	28 (36)	22 (31.4)	7 (70)	0.02
**Other CNS medications:**	53 (66.2)	43 (61.4)	10 (100)	0.003
Benzodiazepines	22 (27.5)	15 (21.4)	7 (70)	0.002
Anti-epileptics	28 (35)	26 (37.1)	2 (20)	0.27
Anti-psychotics	12 (15)	9 (12.9)	3 (30)	0.19
Anti-depressants	9 (11.2)	5 (7.1)	4 (40)	0.01
Analgesics	3 (3.7)	2 (2.9)	1 (10)	0.34
Anti-parkinsonians	2 (2.5)	2 (2.9)	1 (10)	0.34
Vital signs on presentation:				
Systolic blood pressure (mmHg)	137.2 ± 2.8	138.3 ± 3.0	129.0 ± 5.9	0.28
Diastolic blood pressure (mmHg)	79.5 ± 1.6	80.1 ± 1.7	75.3 ± 5.0	0.33
Heart rate (BPM)	89. 5 ± 2.0	88.1 ± 2.0	99.1 ± 6. 9	0.07
O_2_ Saturation% (*n* = 56)	94.3 ± 1.1	95.1 ± 0.6	89. 9 ± 6.4	0.44
ECG abnormalities^a^	8 (10)	6 (8.6)	2 (20)	0.52
Management:				
Activated charcoal	22 (27.5)	17 (24.3)	5 (50)	0.11
Nasogastric tube	26 (33)	19 (27.5)	7 (70)	0.01
Mechanical ventilation	6 (7.5)	2 (2.9)	4 (40)	0.0001
ICU admission	16 (20)	10 (14.3)	6 (60)	0.002
Hospital stay (days)	5.9 ± 1.17	5.4 ± 1.29	8.8 ± 2.1	0.34

*P-value was calculated by t-tests for continuous variables and by Chi-square test for ordinal variables

^a^**ECG abnormalities, including:** Prolonged QTc, T wave changes, AV Block, Atrial Flutter, AF, Bradycardia

**Fig. 2:**
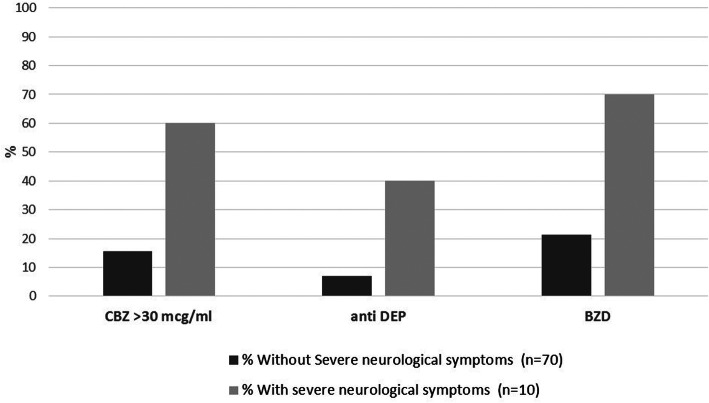
Carbamazepine intoxication- concomitant medications and carbamazepine concentrations among patients with and without severe neurological symptoms. *all *p*-value< 0.05

Concomitant CNS medications were used by 61% of patients without severe neurological symptoms and 100% of patients with severe neurological symptoms (*p* = 0.003). Specifically, higher proportions of benzodiazepines (*p* = 0.002), (odds ratio 8.6, 95% confidence interval: 2.0–37.1, *p* = 0.004) and antidepressants use (*p* = 0.009), (8. 7, 1.8–41.2, *p* = 0.007) were observed (Fig. 2).

### Carbamazepine concentrations and clinical presentation

In patients with severe neurological symptoms, mean carbamazepine level was 40.0  11.07 mcg/ml and 24.39 ± 1.19 mcg/ml in patients without severe symptoms (*p* = 0.19) (Table 2).

Carbamazepine level above 30 mcg/ml was observed in 6 (60%) patients with severe neurologic symptoms, as compared with only 11 (15.7%) patients without severe symptoms (*p* = 0.004), (odds ratio 8.1, 95% confidence interval: 1.9–33.23, *p* = 0.004).

### Management of carbamazepine intoxication

The management of patients is presented in Table 2. Mechanical ventilation was required in 7.5% of the patients and 12.5% required respiratory support. Oxygen saturation was measured in 56 subjects. The mean oxygen saturation in these patients was 94.3% ± 1.1. Among the 10 patients with severe neurological symptoms, mean oxygen saturation was 89.88%, while among patients without severe neurological symptoms it was 95.08%. Cardiac arrhythmia, most commonly sinus tachycardia, was found in 10% of all patients.

Treatment included active charcoal in 28% of patients and gastric lavage in 14%. Clinical monitoring in the ICU was performed in 20% of all patients. The average length of hospital stay in all patients was 5.9 ± 1.2 days.

Among patients with severe neurological symptoms as compared to patients with no severe symptoms, more patients required respiratory support and ICU admission [5 (50%) vs 5 (7.1%) (*p* = 0.002), and 6 (60%) vs. 10 (14.3%) (*p* = 0.002), respectively]. No significant difference was found in the rate of cardiac arrhythmias, cardiovascular indices, or the average duration of hospitalization.

### Multivariate analyses

In order to evaluate the combined contribution of CNS affecting medications on severe neurological presentation, logistic regression was performed including carbamazepine concentration above 30 mcg/ml and the consumption of antidepressants or benzodiazepine, medications that were associated with this outcome in univariate analysis (Table 2).

As multivariate logistic regression with fewer than 5 events per variable frequently results in biased estimates [11], we examined models including benzodiazepines and antidepressants consumption separately, each in combination with carbamazepine concentration above 30 mcg/ml (Table 3).

**Table 3:** Models for the effect of multi drug exposure on severe neurological manifestations during carbamazepine intoxication. Independent variables include carbamazepine concentration > 30 mcg/ml, consumption of antidepressants, or consumption of benzodiazepines

Model	−2 Log likelihood	Variables in model OR (95% Confidence Interval) *p-*value		
		CBZ > 30	AntiDEP	BZD
CBZ > 30	51.87	8.1 (1.9–33.3) *p* = 0.004	–	–
CBZ > 30, AntiDEP	47.35	6. 7 (1.5–29.9) *p* = 0.013	6.6 (1.2–36.5) *p* = 0.029	–
CBZ > 30, BZD	43.20	9.3 (1.9–46.6) *p* = 0.007	–	9.8 (1.9–50.0*p* = 0.006

*CBZ > 30* Carbamazepine concentration above 30mcg/ml

*BZD* Benzodiazepines

*AntiDEP* Antidepressants

In a model including antidepressants use and carbamazepine concentration above 30 mcg/ml (Table 3), antidepressants use increased the odds of severe neurologic manifestations 6.7-fold (1.2–36.5) (*p* = 0.029), and carbamazepine levels higher than 30 mcg/ml increased the odds 6.67- old (95% CI 1.5–29.9) (*p* = 0.013).

In a model including consumption of benzodiazepines and carbamazepine concentration above 30 mcg/ml (Table 3), benzodiazepines use increased the odds of severe neurologic manifestations 9.8-fold (1.9–50.0) (*p* = 0.006) and levels higher than 30 mcg/ml increased the odds of severe neurologic manifestations 9.3-fold (95% CI 1.9–46.6) (*p* = 0.007).

Multivariate logistic regression analysis was performed to identify the effect of CNS affecting medications on ICU admission. In the analysis, carbamazepine concentration above 30 mcg/ml and use of antidepressants increased the odds of ICU admission [(12.5- (3.3–47.5), (*p* = 0.001), and 5.7(1.01–31.7) (*p* = 0.049), respectively], while the use of benzodiazepines was not associated with the odds of ICU admission.

## Discussion

In this study, we found that carbamazepine levels higher than 30 mcg/ml and benzodiazepines and antidepressants exposures were associated with the odds of severe neurological symptoms during carbamazepine intoxication, while ICU admission was associated with carbamazepine levels and antidepressants exposure, but not with benzodiazepines use. This suggests that carbamazepine levels and antidepressants exposure, as compared to benzodiazepines exposure, may have an important role to play in the decision-making process regarding patients’ hospital placement.

Carbamazepine level associated with severe neurological manifestations in our study was 30 mcg/ml, which is lower than the concentration previously reported to be associated with a severe neurologic presentation in adults [3] . This may be related to the high rate of combined drug exposure (66%) in our population, potentially resulting in pharmacodynamic and/or pharmacokinetic interactions with carbamazepine.

Our findings that antidepressants, benzodiazepines use and carbamazepine concentration above 30 mcg/ml were independently associated with severe neurological manifestations support our hypothesis that concomitant CNS affecting medications increase the risk of severe manifestations during carbamazepine intoxication. These findings suggest that multidrug intoxication affects the clinical presentation of carbamazepine intoxication.

Combined intoxication may reduce the threshold for neurological manifestation in various clinical settings. For example, the pharmacodynamic interaction between benzodiazepines and alcohol, resulting in more severe neurological signs even in relatively low benzodiazepines doses, has been previously reported [12]. A pharmacodynamic interaction between carbamazepine and benzodiazepines is supported by shared activity on the GABA-A receptor [13] . Pharmacokinetic factors could also affect our results. Both carbamazepine and benzodiazepines are at least partially metabolized by the same hepatic enzyme, Cytochrome P-450 (CYP3A4), although carbamazepine is also an inducer of this enzyme. Therefore, a competitive inhibition can result in increased plasma concentrations of carbamazepine and/or benzodiazepines.

In contrast to the factors related to severe neurological symptoms, ICU admission was associated with carbamazepine levels and antidepressants use, but not with benzodiazepines use. This suggests that carbamazepine levels and antidepressants use, as compared to benzodiazepines exposure, may have an important role in the decision-making process regarding patients’ hospital placement.

Psychiatric background disorders were associated with higher carbamazepine plasma concentration and severe neurological symptoms. This could be related to high carbamazepine doses used in suicide attempts in this population. However, our findings, that benzodiazepines and antidepressants use were associated with risk for severe neurological manifestation independently from carbamazepine concentration above 30 mcg/ml, suggest that the impact of benzodiazepines and antidepressants use is not the result of higher carbamazepine doses during suicide attempts, and may be mediated by the effects of these drugs in the CNS.

We observed an ethnic difference in the severity of toxicity. We are not aware of a difference in carbamazepine metabolism that could contribute to the difference observed. A careful pharmacokinetic study comparing carbamazepine pharmacokinetics in Arabs and Jews can elucidate this issue.

Our study has several limitations. Our population included patients whose routine monitoring of carbamazepine levels was performed in a hospital laboratory serving both ambulatory and hospital patients, thus it is likely that the study population included more severe cases than the general population of patients treated with carbamazepine.

Since the data collected represent ‘real-life’ conditions during acute intoxication, the timing of carbamazepine ingestion might have not been reliably reported. However, it can be assumed that carbamazepine concentrations were measured close to the peak of symptoms and therefore may correlate with the drug’s peak level. The intake of co-ingestions was based on documentation of medical history and was not analytically confirmed. Since carbamazepine’s active metabolite 10,11epoxide concentration is not routinely measured in our hospital we did not evaluate its effect on the course of clinical symptoms of intoxication.

The true incidence of ECG changes might have been underestimated in our study due to the incomplete documentation and description of ECG findings. Also, GCS [14] or Reed Scale was not always available. Thus, we defined severe neurological manifestations of carbamazepine intoxication according to the presence of major neurological symptoms/signs that reflect severe neurological insult.

## Conclusions

The use of medication combinations in medical therapy and suicide attempts is common, but there is insufficient data regarding their effect on carbamazepine concentration associated with severe neurological manifestation of intoxication. In this study, we found that the use of antidepressants or benzodiazepines independently from carbamazepine concentration above 30 mcg/ml was significantly associated with severe neurological manifestations. This carbamazepine concentration is somewhat lower than the previously reported levels in adults. These results highlight the importance of ascertaining patients’ history such as benzodiazepine and antidepressant use, in addition to carbamazepine blood levels, when evaluating carbamazepine overdose and assessing the need for ICU monitoring in multidrug carbamazepine toxicity.

## Data Availability

The datasets used and/or analyzed during the current study are available from the corresponding author on reasonable request.

## Abbreviations

CNS: Central-nervous-system
ICU: Intensive Care Unit
GCS: Glasgow-coma score

## References

[1] Doyon S (2016). Goldfrank’s Toxicologic Emergencies.48 : Antiepileptics.

[2] Gheshlaghi F, Yaraghi A, Soh EH, Ghoreishi A (2012). Relationship of cardiovascular complications with level of consciousness in patients with acute carbamazepine intoxication. Med Arh.

[3] Hojer J, Malmlund HO, Berg A (1993). Clinical features in 28 consecutive cases of laboratory confirmed massive poisoning with carbamazepine alone. J Toxicol Clin Toxicol.

[4] Spiller HA, Krenzelok EP (1993). Carbamazepine overdose: serum concentration less predictive in children. J Toxicol Clin Toxicol.

[5] Montgomery VL, Richman BJ, Goldsmith LJ, Rodgers GCJ (1995). Severity and carbamazepine level at time of initial poison center contact correlate with outcome in carbamazepine poisoning. J Toxicol Clin Toxicol.

[6] Spiller HA, Krenzelok EP, Cookson E (1990). Carbamazepine overdose: a prospective study of serum levels and toxicity. J Toxicol Clin Toxicol.

[7] Weaver DF, Camfield P, Fraser A (1988). Massive carbamazepine overdose: clinical and pharmacologic observations in five episodes. Neurology..

[8] Schaffer A, Weinstock LM, Sinyor M (2017). Self-poisoning suicide deaths in people with bipolar disorder: characterizing a subgroup and identifying treatment patterns. Int J Bipolar Disord.

[9] COBAS (1998). INTEGRA 700 method manual. Therapeutic Drug Monitoring.

[10] ATC code. https://www.whocc.no/atc_ddd_index.

[11] Vittinghoff E, McCulloch CE (2007). Relaxing the rule of ten events per variable in logistic and cox regression. Am J Epidemiol.

[12] Tanaka E (2002). Toxicological interactions between alcohol and benzodiazepines. J Toxicol Clin Toxicol.

[13] France A, Bagneux X, Depoortere H, Synth P, October R, March A (1995). Modulation antiepileptic of the y-aminobutyric drugs carbamazepine and phenytoin by the.

[14] Reith FCM, Van den Brande R, Synnot A, Gruen R, Maas AIR (2016). The reliability of the Glasgow coma scale: a systematic review. Intensive Care Med.

